# Supplementary material for: The impact of providing hiding spaces to farmed animals: A scoping review
Source: PLoS One. 2022 Nov 28;17(11):e0277665. doi: 10.1371/journal.pone.0277665 (PMC9704605; doi:10.1371/journal.pone.0277665)
Supplement: Attachment — Submitted filename: PONE072322.docx [file pone.0277665.s002.docx]

| Line Number: | Comment/Question: |
| --- | --- |
| 45 | It may be useful to choose to use either ‘welfare’ or ‘well-being’ exclusively in your manuscript. Otherwise, it may be valuable to indicate to the reader that you are using the terms synonymously. |
| 74 | Here and throughout, use past tense to describe actions you took in conducting your review. For example, “To be included, a study must have had at least…” |
| 112-113 | Here and throughout, change actions that were completed in the past to past tense. |
| 127 | Were there specific factors that helped the authors to determine if the overall outcome was positive, negative, or inconclusive/neutral? In its current form, this statement is subjective and may be difficult to replicate. |
| 217-218 | Please provide more explanation of the following statement: “Wild lobsters also make great use of hiding places throughout their lives.” |
| 235 | Consider replacing ‘stage’ with ‘time’ to remain consistent with earlier text. |
| 386 | Omit ‘them’. |
| 426 | Instead of stress in general, is distress a more appropriate term? |
| 478 | If possible, it would be useful to include the number of studies that introduced other enrichment that may be confounding. |

Overall comments: Your scoping review pulls together a growing pool of literature to identify consensus and needs across farmed species. It is a well-written manuscript that warrants publication after some minor editing.
