# Supplementary material for: The impact of providing hiding spaces to farmed animals: A scoping review
Source: PLoS One. 2022 Nov 28;17(11):e0277665. doi: 10.1371/journal.pone.0277665 (PMC9704605; doi:10.1371/journal.pone.0277665)
Supplement: Attachment — Submitted filename: Response to Reviewers.docx [file pone.0277665.s003.docx]

Reviewer 1:

Overall comments:

Your scoping review pulls together a growing pool of literature to identify consensus and needs across farmed species. It is a well-written manuscript that warrants publication after some minor editing.

**Response: Thank you!**

45 It may be useful to choose to use either ‘welfare’ or ‘well-being’ exclusively in your manuscript. Otherwise, it may be valuable to indicate to the reader that you are using the terms synonymously.

**Response: We replaced the term ‘well-being’ with the term ‘welfare’ for improved clarity.**

74 Here and throughout, use past tense to describe actions you took in conducting your review. For example, “To be included, a study must have had at least…”

**Response: Good catch!** **Present tense actions were replaced with past tense actions.**

112-113 Here and throughout, change actions that were completed in the past to past tense.

**Response: Present tense actions were replaced with past tense actions.**

127 Were there specific factors that helped the authors to determine if the overall outcome was positive, negative, or inconclusive/neutral? In its current form, this statement is subjective and may be difficult to replicate.

**Response: More details were added on the process of classifying outcomes as positive, negative, or inconclusive/neutral.**

217-218 Please provide more explanation of the following statement: “Wild lobsters also make great use of hiding places throughout their lives.”

**Response: More details were added to this sentence.**

235 Consider replacing ‘stage’ with ‘time’ to remain consistent with earlier text.

**Response: Good suggestion – ‘stage’ was replaced with ‘time’ to improve consistency throughout the text.**

386 Omit ‘them’.

**Response: Good catch – ‘them’ was omitted.**

426 Instead of stress in general, is distress a more appropriate term?

**Response: We changed lines 544-456 to read “It was commonly concluded that the reduction of abnormal repetitive behaviours was because hiding spaces helped reduce negative affective and physiological states that are presumed to be negative, such as perceived boredom, distress, or fear.”**

478 If possible, it would be useful to include the number of studies that introduced other enrichment that may be confounding.

**Response: The number of studies that provided hiding places in combination with other enrichment items was provided on line 568 (37 out of the 151 papers included in the review).**

**Reviewer 2:**

Overall comments:

This field of research is a huge undertaking, and the paper does a good job of describing the current literature. The topic is of importance to the scientific community and the results are novel. In general, with so many species I think it may be easy to get lost in the weeds. Something I think could strengthen this paper is a description of the function of hiding behavior in the introduction (or elsewhere if there is a better fit). For example, cattle separate from the herd and use environmental coverage at calving in semi-natural settings, pigs build themselves a nest before farrowing, and mink/foxes/rabbits live in burrows or dens. And then why is providing hiding opportunities for farmed species important? This could also use additional set-up in the introduction. Specific comments are below.

**Response: We thank the reviewer for their very thorough and thoughtful comments! We agree that the function of hiding behavior is a critical component of this review, so we have added** **background information on the natural hiding behaviour of wild animals on Lines 49-76. We have also added more information about why this is important for animal welfare on Lines 77-80.**

The abstract describes the methodology well but in general is missing a set-up of why opportunities to hide is important for farm species. Similarly, the rationale for describing negative/neutral outcomes is unclear without first describing if hides benefit animals.

**Response: We agree and have restructured the beginning of the abstract to now read: “Many wild animals will perform hiding behaviours for a variety of reasons, such as evading predators or other conspecifics. Unlike their wild counterparts, farmed animals are often housed in relatively barren environments without the opportunity to hide. Researchers have begun to study the impact of access to hides in farmed animals, including possible effects on animal welfare.” We are limited in word count for the abstract, so we hope that this concise set-up helps provide context for the review. We also did not want to assume that hides were good for animals until we completed the review (as this was the purpose of objective 3), so we have left this out and kept it more open.**

Specific comments are below.

15-16: It is unclear here why the link is being made or how hiding affects farmed animals

**Response: We have changed the wording of the abstract based on the reviewers earlier comment.**

17-20: I’m curious about the use of the term “hides”. It isn’t defined clearly in the abstract. Could a better term be “hiding spaces” if “hide” isn’t defined right away?

**Response: Good point – the text has been modified to indicate that the terms “hiding space” and “hides” are being used synonymously.**

28-31: What do the % values refer to?

**Response: Text has been added to clarify that the % values are indicating the % of papers included in the review. As we are limited on word count, we added this to the first % and left it off of the rest.**

32-33: Why highlight the inconclusive/negative outcomes from the review without the positive?

**Response: Due to a limited word count we have opted to make this summary shorter so that Line 34-36 now reads: “Hides had a generally positive impact on animals, but more research is encouraged for under-represented species.”**

33-35: To include this summary consider first describing the species, age, and health status results found in the scoping review

**Response: This sentence was again shortened to meet the word count – we have opted to leave this more open-ended and explain the gaps more in the discussion. Line 34-35 now reads: “Hides had a generally positive impact on the animals included in this review; more research is encouraged for under-represented species.”**

45: Consumers or the public?

**Response: Good comment – we changed the term “consumer” to the “citizen” to better capture the meaning from the cited papers.**

46-48: This sentence is somewhat unclear in the direction of causation. It seems like naturalness in production is a problem because opportunities to perform natural behaviors aren’t provided and may be motivated to do so

**Response: The wording of this sentence was changed for improved clarity.**

49-55: The topic of hiding in all farm species is cumbersome but I think it would be worthwhile to expand on the function of hiding behavior in natural or semi-natural settings and why this topic is important enough for a scoping review (i.e., some animals are highly motivated to hide during specific events)

**Response: We agree with this comment and have added a paragraph describing the possible functions of hiding behaviors in species that are related to the farmed species included in this review (Lines 49-76).**

56: Again, why is the research in this field of study increasing?

**Response: Additional information was added to describe how allowing farmed animals to perform natural behaviours can benefit welfare. Lines 77-82 now reads: “Allowing farm animals the opportunity to perform natural behaviours, such as hiding, is an important component of animal welfare for many stakeholders. Moreover, allowing the performance of some natural behaviors may also improve other aspects of welfare, such as affective states and biological functioning (ref). Because of these potential benefits, there has been increasing research on allowing farmed animals to perform natural hiding behaviours, despite few conventional management systems that currently allow for this behaviour.”**

59-63: I would suggest defining the term “hide” in the introduction prior to stating the objectives. Not all hiding spaces are referred to as hides in the literature (e.g., nest boxes for hens)

**Response: Good point here, we added a description of what we considered a hide into this section.**

65: In general, the methods section is a little bit murky. I would suggest making the definitions for screening/inclusion for clear cut. For example, potentially identify the questions each title/abstract was assessed for, then what criteria made it an inclusion/exclusion to full text criteria. You may consider using bullet points or a numbered list for clarity

**Response: We have changed this section so that our inclusion criteria is now a numbered list to help for clarity.**

97: How many results were found for each search term?

**Response: We clarified in our methods section that the searches were ran as a single search for all search terms. Because of this, results for each individual search term are not available.**

114: Was a full text screening performed after the title/abstract screening?

**Response: Yes – we added details about our full text screening process.**

120: Suggest leaving the number of papers out because it isn’t presented until the results section

**Response: The total number of papers was removed from this line.**

120-121: What does “according to the aims of the paper” mean? This could use additional detail

**Response: The wording of this sentence was altered to improve clarity so that lines 151-152 now read: “The 151 papers included in the final review were coded to facilitate analysis of the aims of this paper.”**

122: The reason for animal categorization needs to be defined. How/why you decided on specific categories should really be described. For example, why not categorize animals into “mammal, bird, fish, etc.” or “herbivore, omnivore, carnivore”? If there are hiding behaviors specific to each group you have identified, make sure its clear in the methodology. Also, crustacean should be reflected in Figure 2

**Response: Details about how and why we selected these categories have been added into this section, so that Lines 156-162 now read: “We chose to use these specific “animal types” due to their relevance to farm animal industries. We also opted to further group them into “animal categories” based on biological and behavioural similarities; for example, we used the broader category of ungulate to represent cattle, deer, pigs, sheep, and goats because these animals have similar natural histories. However, rabbits and lobsters were the only animal types found in our search that fit in the small herbivore and crustacean categories.” The figures have also been changed to be aligned with the changes made in this section.**

Line 125: Was sample size included?

**Response: Sample size may be relevant to include for a systematic review; however, we chose not to include sample size because this is a less detailed review.**

126-127: The terms “positive, negative, neutral” are somewhat subjective. Its unclear if the authors have defined the impacts on the animals or they are reporting what the authors of the original articles determined were “positive/negative/neutral”. A better way to report outcomes may be by creating a definition for these terms first before describing the categorization. E.g., an outcome was determined to be “positive” if it reduced disease, was preferred by animals, or reduced the performance of abnormal behaviors

**Response: We have added much more details on the process of classifying outcomes as positive, negative, or inconclusive/neutral.**

131-140: Make sure all these criteria are outlined in the methods. This is the first time full text review and hand-searching papers is discussed

**Response: Details on the full text review and hand searching were added to the methods section.**

142: Why not report year of publication and country of study? These were included in the categorization (line 122)

**Response: Good point - we added the range of publication years in the results section to demonstrate that research in this area is increasing over time. We removed country of study since, while interesting, we don’t feel it contributes to our aims.**

182-184: What does this mean? If a study had multiple outcomes (e.g., behavior and production) each outcome was included in the table? Additionally, it may be helpful to describe the # of papers that had each of the 7 categorized outcomes

**Response: We have added language at the start of this section to describe how multiple outcomes were counted (lines 248-249). We also added a description of the number of papers that reported each outcome category (lines 260-266).**

190/Table 2: Crustaceans should either be lobsters/crustaceans throughout to be consistent. What each of these categories mean is unclear at this point, I’m hoping the definitions/descriptions will be elaborated on in the discussion.

**Response: Further detail has been added into the paper to improve the meaning of our categories. The crustacean/lobster inconsistencies have been corrected.**

General comment on results: The results so far are interesting but feel as though they are lacking some meaning. I would suggest trying to dig in to the function of hiding behavior. Potentially this could be done by adding another table like table 2 with “context of hide” as the other category

**Response: We like this idea and have added the context of hide provision into table 2 for each animal category.**

207: Domestic animal behavior research?

**Response: Good suggestion, we modified the wording of this sentence for improved clarity.**

211: 6-13 studies per species listed?

**Response: Yes, we added clarification to the text.**

213: The 1 to 5 studies is confusing, like above, it may be clearer to provide the number of % of studies after each species

**Response: We modified the wording to include the number of studies after each animal type for improved clarity.**

225: This is the first time these species are mentioned. They should be described in the methods and/or results

**Response: These species were mentioned in the results section on lines 221-223: “There were 6 animal types included in the initial literature search for which no results were found: bison, elk, alpacas, llamas, horses, and donkeys.”**

258: There are 2 published studies on the hiding behavior of ewes at parturition in “cubicles” - Use of Lambing Cubicles and the Behavior of Ewes at Parturition (Gonyou and Stookey, 1983) and Behavior of parturient ewes in group lambing pens with and without cubicles (Genyou and Stookey 1985)

**Response: Thank you for the excellent suggestion of additional relevant papers, we have added these papers into the review.**

332-335: The cattle described Lidfors (71) and Edwards papers (73) separated from other cattle and sought more ‘covered’ areas around parturition. However, neither of these groups of animals used man-made “hiding spaces” to calve. Here, and elsewhere as it applies, it may be more correct to describe the animal behavior rather than the defined environment

**Response: We like this idea and have added clarification to what kinds of hiding spaces were used by these animals and the specific behaviors that were performed.**

354: What kind of injury?

**Response: Text was added to clarify that this paper explored disbudding-related injuries.**

433: I’m curious about the injuries described here. Are these injuries from the environment, self-mutilation, aggressive interactions between animals, or something else?

**Response: More detail about the types of injuries was added.**

450: Affective state needs to be defined somewhere

**Response: Good suggestion, we added in a brief definition of affective state in the introduction.**

Figure 1: Does “Records screened” refer to the title/abstracts screened (same for records excluded)? Was a full text screening performed? And the final box, what is “reports of included studies”

**Response: In Figure 1, records screened refers to the title and abstract screening, and reports assessed refers to the full text review. The process for the title/abstract screening and the full text review have been clarified in the methods. “Reports of included studies” should be the same number as the number as “studies included in the review”, since there were no papers found that reported the same study data. This has been corrected in the figure.**

Figure 2: Y-axis should be “number of studies per species”. All the species have a larger umbrella/category, would suggest giving lobsters and rabbit a category too to define why they don’t fit into any of the other categories.

**Response: The axis and umbrella categories were corrected, and an explanation for the lack of greater categories for rabbits and lobsters was added into the methods.**
