# Supplementary material for: The impact of providing hiding spaces to farmed animals: A scoping review
Source: PLoS One. 2022 Nov 28;17(11):e0277665. doi: 10.1371/journal.pone.0277665 (PMC9704605; doi:10.1371/journal.pone.0277665)
Supplement: Attachment — Submitted filename: Response to Reviewers (1).docx [file pone.0277665.s004.docx]

**The paper has improved substantially. Thank you to the authors for such thorough revisions. There are only a few minor comments to address:**

**58 – 60: Does this mean mink and foxes also hide their offspring to avoid predation and infanticide?**

Answer: Yes, this was the intended meaning. We slightly modified the wording to improve clarity.

**164: In this paper, there are multiple references to “themes” or “themes that emerged”. Was there a specific thematic analysis applied to the papers to determine themes present in the reviewed papers? Or were themes determined by the greatest % of outcomes? You may consider explaining how “themes” were defined**

Answer: We did not perform a thematic analysis, and we modified the wording of this sentence to better reflect our methodology.

**226 – 228: Could the “these animals were represented” be changed to something more specific to the figure? Ideally, someone should be able to interpret a figure with only the figure description without needing to read the paper**

Answer: We appreciate this suggestion, and we modified the wording of the figure to be more comprehensible as a stand-alone description of the figure.

**240: Same comment as above regarding the figure description**

Answer: We similarly modified the wording of this figure.

**290: The reference to chickens being one of the ‘most widely used food animals’ could benefit from some content/explanation. Are they common in Canada, North America, or globally? Additionally, is the reference to broilers or laying hens**

Answer: Clarity was added into the sentence for both questions.

**295: There are 2 commas between “turkeys” and “and”**

Answer: Thank you, this has been resolved.

**295 – 297: In terms of a lack of research, is it specific to animals in natural or farmed settings?**

Answer: We were referring to lack of research in farmed settings, this has been clarified.

**302: Should the period be after 40?**

Answer: The unneeded references after the period were removed, resolving the seemingly incorrect period placement.

**315-316: Should the number of parturition/oviposition papers be the same? I.e., 80/151 and 63/84**

Answer: Thank you for catching this, they should be the same. The numbers 80/151 were corrected to 84/151.

**477-479: Missing a reference?**

Answer: Good catch – the reference was added in.

**Many of the paragraphs in the discussion end with – more work is need (302-303, 310-312, 347-349, 406-408, 432-433, 455-456, 461-462, 471-472, 480-481, 490-491, 507-508, 527-529, 540-542, 562-564). It appears one of the main conclusions of the review is that many areas of study need additional research. However, I’m not sure it warrants stating so frequently and some of the individual paragraph summaries could be more insightful to the topic at hand.**

Answer: We appreciate the identification of repetitive areas of the manuscript and have reviewed the highlighted portions. We are choosing to retain many of these sentences as identification of gaps in the literature was one of our main objectives; however, we modify or eliminate some of these phrases (lines 306-307, 494-495, and 544-546 in the manuscript with tracked changes).

**580: Does chickens refer to broilers or laying hens?**

Answer: This was referring to laying hens, and this was added into the sentence for better clarity.
